# Epidemiological profile of inflammatory bowel disease in Caxias do Sul, Brazil: a cross-sectional study

**DOI:** 10.1590/1516-3180.2020.0179.R2.10092020

**Published:** 2020-11-13

**Authors:** Vincent Marin Dall'Oglio, Rafael Sartori Balbinot, Ana Laura Facco Muscope, Mateus Dal Castel, Thianan Ricardo Souza, Renan Souza de Macedo, Thanize Barbosa de Oliveira, Raul Angelo Balbinot, Silvana Sartori Balbinot, Eduardo Brambilla, Jonathan Soldera

**Affiliations:** I MD. Resident, Department of Internal Medicine, Hospital Geral de Caxias do Sul, Caxias do Sul (RS), Brazil; Resident, Department of Gastroenterology, Hospital de Clínicas de Porto Alegre, Porto Alegre (RS), Brazil.; II MD. Physician, School of Medicine, Universidade de Caxias do Sul (UCS), Caxias do Sul (RS), Brazil; Resident, Department of Internal Medicine, Universidade Federal de Ciências da Saúde de Porto Alegre (UFCSPA), Porto Alegre (RS), Brazil.; III MD. Physician, School of Medicine, Universidade de Caxias do Sul (UCS), Caxias do Sul (RS), Brazil.; IV MD. Physician, School of Medicine, Universidade de Caxias do Sul (UCS), Caxias do Sul (RS), Brazil.; V MD. Physician, School of Medicine, Universidade de Caxias do Sul (UCS), Caxias do Sul (RS), Brazil; Resident, Department of Pediatrics, Universidade Federal de Ciências da Saúde de Porto Alegre, Porto Alegre (RS), Brazil.; VI MD. Physician, School of Medicine, Universidade de Caxias do Sul (UCS), Caxias do Sul (RS), Brazil.; VII MD. Physician, School of Medicine, Universidade de Caxias do Sul (UCS), Caxias do Sul (RS), Brazil.; VIII MD, MSc, PhD. Titular Professor, Department of Clinical Gastroenterology, School of Medicine, Universidade de Caxias do Sul (UCS), Caxias do Sul (RS), Brazil.; IX MD, PhD. Titular Professor, Department of Clinical Gastroenterology, School of Medicine, Universidade de Caxias do Sul (UCS), Caxias do Sul (RS), Brazil.; X MD, MSc. Proctologist and Associate Professor, Department of Clinical Gastroenterology, School of Medicine, Universidade de Caxias do Sul (UCS), Caxias do Sul (RS), Brazil; Associate Member, Grupo de Estudos da Doença Inflamatória Intestinal do Brasil (GEDIIB), Sao Paulo (SP), Brazil.; XI MD, MSc. Associate Professor, Department of Clinical Gastroenterology, School of Medicine, Universidade de Caxias do Sul (UCS), Caxias do Sul (RS), Brazil; Doctoral Student: Pathology, Universidade Federal de Ciências da Saúde de Porto Alegre (UFCSPA), Porto Alegre (RS), Brazil; Associate Member, Grupo de Estudos da Doença Inflamatória Intestinal do Brasil (GEDIIB), Sao Paulo (SP), Brazil.

**Keywords:** Inflammatory bowel disease, Crohn disease, Colitis, ulcerative, Infliximab, Adalimumab, Latin America, South America, Mesalamine, Indeterminate colitis, Epidemiology of inflammatory bowel disease

## Abstract

**BACKGROUND:**

Inflammatory bowel diseases affect mostly young patients and have a huge impact on their quality of life and growing treatment costs. Currently, there are few Brazilian studies concerning their epidemiological profile.

**OBJECTIVE:**

The aim of this study was to describe the regional clinical and epidemiological profile of these pathological conditions in Caxias do Sul, Brazil.

**DESIGN AND SETTING:**

Cross-sectional study in Caxias do Sul (RS), Brazil.

**METHODS:**

A search for patients was conducted in the municipality's special medications pharmacy using the International Classification of Diseases, and medical records were manually reviewed for data collection. Sixty-seven patients were included.

**RESULTS:**

The patients’ mean age was 46.5 years and females predominated (71.6%). Ulcerative colitis was the most prevalent disease (70%) and Montreal E3 was the most prevalent presentation. The mean age at diagnosis was 39 years. Most patients had recently undergone colonoscopy (67%). Only five patients (7.4%) had records of hospital admission due to the disease, while 12 (18%) underwent a surgical procedure during follow-up. Sixty patients (89.5%) were using aminosalicylates, while less than one fifth were using immunosuppressants or immunobiological drugs: 19.4% and 14.9%, respectively.

**CONCLUSION:**

The profile of inflammatory bowel disease patients in this region of Brazil is similar in some characteristics to other published Brazilian data, although it differs in others such as higher frequency of pancolitis. A prospective study on these patients is planned in this region, in order to improve the data quality.

## INTRODUCTION

Inflammatory bowel diseases comprise two major disorders: ulcerative colitis and Crohn's disease. These two pathological conditions have distinct and, at the same time, overlapping clinical characteristics, which might occasionally lead to indeterminate classification.

Globally, the regions with the highest prevalence of these conditions include North America and Northwest Europe. Even with a significant increase in incidence in the last couple of decades, Brazil is still considered to be a low-prevalence country. Nevertheless, there is a lack of clinical-epidemiological studies about inflammatory bowel disease in South America.^1^^,^^2^ The factors that have been responsible for the remarkable increases in incidence of these diseases, especially in industrialized countries, are still unknown. These increases may have been related to changes in hygiene habits or diet, transition of the population to urban areas or improvements in diagnostic methods.^3^ It is also possible that diagnosing of inflammatory bowel disease might suffer from underreporting; hence, it is not a disease with compulsory notification in Brazil.^1^

Despite the low mortality rates associated with inflammatory bowel disease, it has a high burden on the private and public health systems, given that it mostly affects young people, especially those of working age. In addition, its chronic profile, with remissions and exacerbations, has a high impact on patients’ quality of life relating to psychological, professional and social matters, which consequently increases healthcare system costs and work incapacity.^4^

Although the pathogenesis and etiology of ulcerative colitis and Crohn's disease remain unknown, it is believed that genetically predisposed individuals are exposed to environmental factors that trigger the disease. This causes a chronic auto-inflammatory process that usually presents with periods of remission and recurrence.^5^

The clinical manifestations of inflammatory bowel disease can vary greatly. The most common symptoms are diarrhea, abdominal pain and rectal bleeding. However, these symptoms can be manifested in other highly prevalent conditions in Brazil, such as bacterial, viral and parasitic intestinal infections. Moreover, inflammatory bowel disease also has extra-intestinal manifestations, generally in the liver, skin, entheses and eyes, which might sometimes precede the intestinal manifestations. Nutritional disorders such as protein-calorie malnutrition, vitamin deficiency and trace elements can also occur.^4^^–^^6^

There is no gold-standard method for diagnosing inflammatory bowel disease. The diagnosis is made from a combination of clinical, endoscopic, radiological, serological and histological findings. However, these methods may not be enough for the diagnosis. In such cases, it is necessary to monitor and observe the natural history of the disease.^4^^–^^6^

Currently, treatments aim to reduce not only the symptoms, but the inflammatory process as well, so as to prevent potential complications. The modality of treatment is based on an assessment that defines degrees of severity and region of involvement. The pharmacological therapy includes anti-inflammatory drugs (salicylate in doses ranging from 2.4 g to 4.8 g daily and corticosteroids, generally prednisone, at a starting dose of 0.5 mg/kg to 1 mg/kg daily) and immunosuppressants (azathioprine 2-2.5 mg/kg/day, 6-mercaptopurine 1.5-2 mg/kg/day and methotrexate 25 mg intramuscularly per week). Recently, use of antitumor necrosis factor alpha (infliximab 5 mg/kg/dose or adalimumab 40 mg) or anti-integrin (vedolizumab 300 mg) has also started. Surgical approaches are reserved for selected patients, such as those who do not respond well to clinical therapy or who present complications (such as hemorrhage, obstruction, intestinal perforation and toxic megacolon).

## OBJECTIVE

The aim of this study was to evaluate and characterize the clinical and epidemiological profile of patients with inflammatory bowel disease in the city of Caxias do Sul (RS), Brazil.

## METHODS

This was a cross-sectional study investigating the profile of patients with inflammatory bowel disease, based on a review of medical records. Data were collected during 2016, through reviewing the medical records at a healthcare center in Caxias do Sul and at the gastroenterology and proctology outpatient clinic of a private university in Caxias do Sul. Both of these centers are referral locations for treatment of inflammatory bowel disease within the Brazilian National Health System (Sistema Único de Saúde, SUS). These centers cover an area of 49 municipalities that have an estimated population of 1,079,601 inhabitants, according to the 2010 population census.

This study was approved in October 2016 by a local ethics committee, under protocol number 57569816.1.0000.5341. Since this study only involved reviewing medical charts, the ethics committee exempted the researchers from the necessity for a consent form.

An active search for patients presenting conditions compatible with the International Classification of Diseases (ICD-10) codes for inflammatory bowel disease (K50 and K51) was conducted. The search was conducted in the municipality's special medications pharmacy and the patients included were living in Caxias do Sul and were using any of the following drugs (all of them supplied through SUS): mesalamine 2 g to 4 g daily, sulfasalazine 2 g to 4 g daily, azathioprine 2-2.5 mg/kg/day, adalimumab 40 mg and infliximab 5 mg/kg/dose. The patients included needed to be under active follow-up in a clinic, have a confirmed diagnosis and have a record of recent prescription refill. Patients younger than 16 years old and those whose medical records could not be accessed, were incomplete or did not exist were excluded from the study.

Physical medical records were analyzed manually and data were gathered in relation to each patient. Type and presentation of inflammatory bowel disease were defined not through the ICD code, but through review of the medical records. Data regarding patient symptoms, treatment, risk factors, extra-intestinal manifestations, examinations and procedures were gathered as described in their respective charts.

It had been planned to analyze Harvey-Bradshaw scores for the severity of Crohn's disease and Mayo Clinic scores for ulcerative colitis. Unfortunately, because of a lack of clinical data in the charts for completing the data for these scores, it was not possible to do this analysis. Data regarding the phenotype of Crohn's disease was not found to be reliable and therefore was not collected.

The statistical analysis on the data consisted of presentation of percentages for qualitative variables, and simple frequencies, averages and standard deviations for quantitative variables. These analyses were done using the IBM SPSS statistical software, version 15.0 (SPSS Inc., Chicago, United States).

## RESULTS

**Figure 1:**
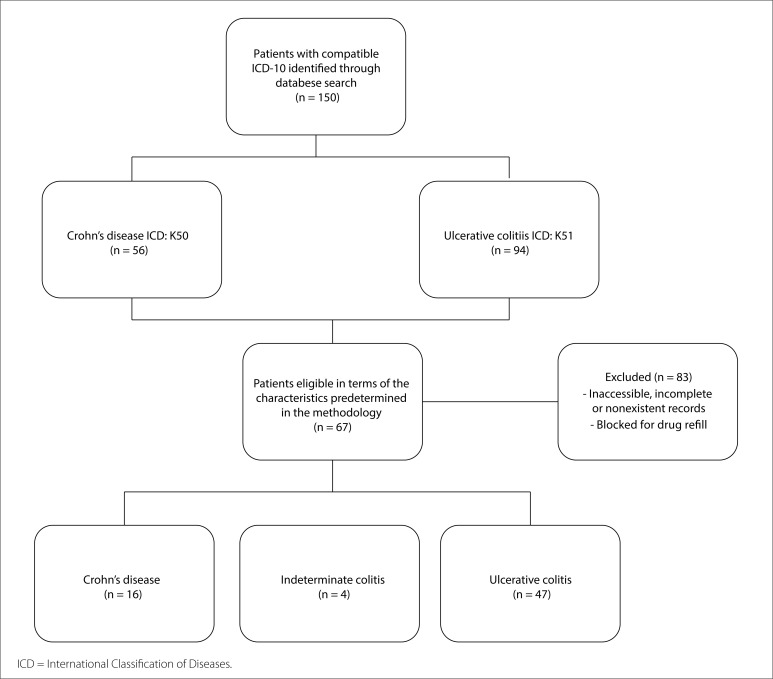
Flowchart for patient selection.

In the initial analysis (Figure 1), 150 patients with current follow-up presenting diseases compatible with ICD-10 codes K50 and K51 were found: 94 with ulcerative colitis and 56 with Crohn's disease. Among these, 85 (56%) were under follow-up at the outpatient clinic of the private university in Caxias do Sul, while the remaining 65 (44%) were attended at the specialized healthcare center. Considering the whole sample, 22 patients (14.6%) were undergoing treatment supervised by a gastroenterologist, while 128 (85.4%) were under the care of a proctologist. Eighty-three patients were excluded from the study due to non-accessible records and/or blocked registrations for medication refill at the specialty center and no further data were available for collection. Therefore, the final number of patients included in the study whose data were gathered from their medical records was 67.

**Figure 2:**
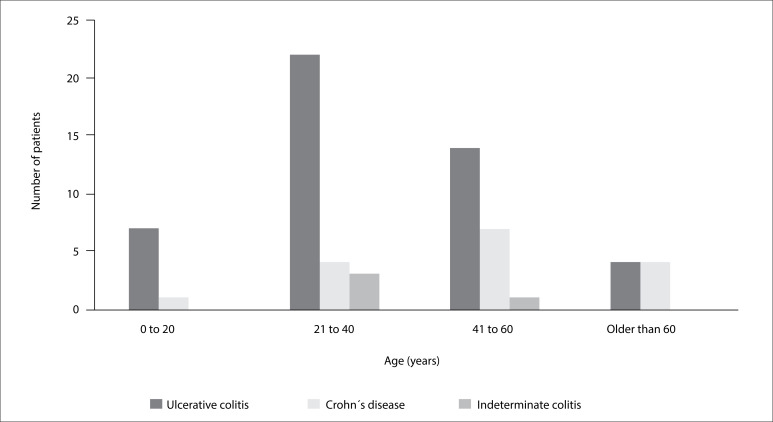
Age at the time of diagnosis.

The main clinical characteristics of the population are described in Table 1. The mean age was 46.5 ± 16.2 years, with a predominance of females (71.6%). Ulcerative colitis was the most common disease, presented by 47 patients (70.2%), while 16 (23.8%) had Crohn's disease and four cases (6%) were undetermined. The mean number of years with the disease was 7.48 ± 6 years. The mean age at diagnosis was 39.1 ± 15.5 years, and the peak incidence was in the age range of 20-40 years (46.2%) (Figure 2).

**Table 1:** Characteristics of the study population according to type of disease

Patients’ characteristics		All patients (n = 67)	Crohn's disease (n = 16)	Ulcerative colitis (n = 47)	Indeterminate colitis (n = 4)
**Gender**					
	Male	19 (28.4%)	6 (37.5%)	11 (23.4%)	2 (50%)
	Female	48 (71.6%)	10 (62.5%)	36 (76.6%)	2 (50%)
**Age (years)**		46.5 ± 16.2	41.1 ± 16.7	47.8 ± 15.1	41.3 ± 10.9
**Period of evolution (years)**		7.4 ± 6	9.4 ± 7.1	6.9 ± 5.5	5 ± 3.3
**Age at diagnosis (years)**		39.1 ± 15.5	31.7 ± 15.1	40.9 ± 13.6	36.3 ± 9.8
**Presentation**					
	Pancolitis	18 (26.8%)	3 (18.8%)	13 (27.7%)	2 (50%)
	Proctosigmoiditis	11 (16.4%)	1 (6.3%)	9 (19.1%)	1 (25%)
	Proctitis	10 (15%)	1 (6.3%)	9 (19.1%)	0
	Left hemicolitis	9 (13.4%)	3 (18.8%)	6 (12.8%)	0
	Segmental colitis with ileitis	4 (6%)	3 (18.8%)	1 (2.1%)	0
	Pancolitis with terminal ileitis	3 (4.5%)	2 (12.5%)	1 (2.1%)	0
	Terminal ileitis	2 (3%)	2 (12.5%)	0	0
	Not available	10 (15%)	1 (6.3%)	8 (17%)	1 (25%)
**Last colonoscopy**					
	2016	21 (31.3%)	7 (43.8%)	12 (25.5%)	2 (50%)
	2015	25 (37.3%)	6 (37.5%)	19 (40.4%)	0
	2014	9 (13.4%)	1 (6.3%)	8 (17%)	0
	2013	3 (4.5%)	0	2 (4.3%)	1 (25%)
	2012	3 (4.5%)	0	3 (6.4%)	0
	Before 2011	6 (9%)	2 (12.5%)	3 (6.4%)	1 (25%)
**Previous hospitalizations due to disease complications**					
	1	2 (3%)	0	2 (4.3%)	0
	2	1 (1.5%)	0	0	1 (25%)
	3	1 (1.5%)	1 (6.3%)	0	0
	4	1 (1.5%)	1 (6.3%)	0	0
	None	62 (92.5%)	14 (77.4%)	45 (95.7%)	3 (75%)
**Surgical procedures performed to manage the disease**					
	Total colectomy	4 (6%)	1 (6.3%)	3 (6.4%)	0
	Hemicolectomy	3 (4.5%)	3 (18.8%)	0	0
	Anal surgery	3 (4.5%)	3 (18.8%)	0	0
	Proctosigmoidectomy	1 (1.5%)	1 (6.3%)	0	0
	Enterectomy	1 (1.5%)	1 (6.3%)	0	0
	No procedure	55 (82%)	7 (43.8%)	44 (92.6%)	4 (100%)
**Bowel evacuations per day**					
	1 to 3	39 (58%)	7 (43.8%)	30 (63.8%)	2 (50%)
	4 to 6	9 (13.4%)	5 (31.3%)	4 (8.5%)	0
	More than 7	5 (7.4%)	2 (12.5%)	3 (6.4%)	0
	Not available	14 (20.8%)	2 (12.5%)	10 (21.3%)	2 (50%)
**Smoking**					
	Yes	5 (7.5%)	0	5 (10.6%)	0
	No or not available	62 (92.5%)	16 (100%)	42 (89.4%)	4 (100%)
**Extraintestinal manifestations**					
	Hematological	6 (9%)	1 (6.3%)	5 (10.6%)	0
	Osteomuscular/articular	3 (4.5%)	1 (6.3%)	1 (2.1%)	1 (25%)
	Dermatological	1 (1.5%)	0	1 (2.1%)	0
	None or not available	57 (85%)	14 (87.5%)	40 (85.1%)	3 (75%)
**Medications in use for treatment**					
	Salicylates	60 (88%)	10 (62.5%)	42 (89.4%)	3 (75%)
	Mesalamine	54 (80.5%)	10 (62.5%)	39 (83%)	3 (75%)
	Sulfasalazine	6 (7.5%)	0	3 (6.4%)	0
	Prednisone	8 (12%)	1 (6.3%)	6 (12.8%)	1 (25%)
	Azathioprine	13 (19.4%)	6 (37.5%)	7 (14.9%)	0
	Immunobiological drugs	16 (23.4%)	8 (50%)	8 (17%)	0
	Infliximab	8 (11.7%)	4 (25%)	4 (8.5%)	0
	Adalimumab	8 (11.7%)	4 (25%)	4 (8.5%)	0
	Metronidazole	1 (1.5%)	0	1 (2.1%)	0

The most common presentation was pancolitis in 18 patients (26.8%), followed by proctosigmoiditis in 11 (16.4%), proctitis in 10 (15%), left hemicolitis in 9 (13.4%), segmental colitis with ileitis in 4 (6%), pancolitis with ileitis terminal in 3 (4.5%) and terminal ileitis alone in 2 (3%). For 10 patients (15%), there was no record of the site of involvement. The majority of the sample (45 patients; 67%) had undergone colonoscopy within the last two years (2015 and 2016) and only seven patients (around 10%) had not undergone colonoscopy in the last five years. Five patients (7.4%) had histories of hospital admission due to some complication of the disease: two patients with one hospitalization, one with two, one with three and one with four. At some time during the evolution of the disease, 12 patients (18%) needed some type of surgical procedure: four of them underwent total colectomy (6%), three had hemicolectomy (4.5%), three had anal surgery (4.5%), one (1.5%) had rectosigmoidectomy and one (1.5%) had enterectomy.

At the time of the last medical appointment, more than half of the patients (39; 58%) were experiencing one to three bowel movements per day, while nine (13.4%) had four to six bowel movements per day and five (7.4%) had more than seven bowel movements per day. There was no record of the number of bowel movements in the cases of 14 patients (20.8%).

The great majority of the patients (62; 92.5%) were nonsmokers and only five (7.5%) were active smokers. In addition, a large proportion of the patients did not have any extraintestinal manifestations (57; 85%), while six (9%) had manifestations of hematological origin (anemia of chronic disease), three (4.5%) had manifestations of osteomuscular/articular etiology and a single patient (1.5%) had dermatological manifestations.

Regarding management, 60 patients (89.5%) were using aminosalicylates, and the most common of these was mesalamine (80%). Only eight (12%) were using oral corticosteroids, which in all cases were exclusively prednisone/prednisolone. Part of the sample was using immunosuppressants. Thirteen patients (19.4%) were using azathioprine. Only one patient (1.5%) was continuously using an antimicrobial, which was metronidazole. Among the Crohn's disease patients, eight (50%) were using immunobiological drugs: adalimumab in four cases and infliximab in the other four cases. Among the ulcerative colitis patients, eight (17%) were using immunobiological drugs: adalimumab in four cases and infliximab in the other four cases.

In the combined analysis, 49 (73.1%) were seen to be undergoing monotherapy and the most common drug was aminosalicylate (75.5%). Fourteen patients (19.4%) were using two drugs and the most common combination was salicylate in association with corticoid, immunosuppressive or biological. Only four patients (6%) were using three drugs and in all of these cases, this comprised salicylate in association with immunosuppressives and immunobiological drugs.

## DISCUSSION

Epidemiological studies on inflammatory bowel diseases in Brazil are few in number and limited in extent. This is due to the significant problems with data record systems that exist in this country. As a result, information on the incidence and prevalence of these diseases are unavailable, although small local studies have identified that their incidence is increasing, particularly with regard to Crohn's disease, in comparison with ulcerative colitis.^7^^–^^9^

In the present study, data from the two referral services for follow-up and treatment of inflammatory bowel disease in the region were gathered. Despite the difficulties encountered in collecting the data and the lack of information in the medical records, this study provides important information on the regional profile, which, until now, has been largely unknown. We found higher prevalence of these diseases among females, a finding similar to what has been reported in other Brazilian studies.^8^^–^^10^ On the other hand, it has been observed in studies conducted in other countries that ulcerative colitis is predominantly found in males and Crohn's disease is predominantly found in females.^11^^,^^12^

The peak age at diagnosis in the present study was within the 20 to 40-year age group, which was already well established in the literature. However, we did not observe any second peak, as previously described in other studies, which usually occurs over the age of 50 years.^13^ These findings are compatible with the data from a recently published systematic review, which found that the prevalence of inflammatory bowel disease has been increasing in Latin America and Carribbean.^14^

Ulcerative colitis was more prevalent than Crohn's disease, which is consistent with previous data from other countries, but discordant with previous Brazilian studies.^1^^,^^7^ Unlike in some older Brazilian case series, which showed predominance of proctosigmoiditis and left hemicolitis,^7^ pancolitis predominated in the present study. This finding was similar to the epidemiological profile observed in a study carried out in a municipality in the state of Santa Catarina in 2011.^9^ One fifth of the sample of the present study had more than three evacuations per day, thus possibly indicating greater severity of disease activity. This subgroup of the sample, in its entirety, was already under treatment with azathioprine and/or immunobiological drugs.

The most common treatment was monotherapy. This was probably because of the mild-to-moderate condition of the disease, better adherence to treatment and reduced adverse effects. The preference for mesalamine was likely due to its availability and the fewer side effects associated with this treatment. The use of immunobiological drugs was three times higher among patients with Crohn's disease, probably due to the many possible complications associated with this disease and the patients’ better response to this therapy. Surgical treatment was necessary for 18% of the patients, which is generally expected for inflammatory bowel disease. Although medical treatment has advanced, a large proportion of patients will still require surgery.^15^^,^^16^

One of the strengths of the present paper is that it reports on a locality where the realities for such patients were unknown. One limitation to this study is its retrospective nature, given that it analyzed medical records. Therefore, it is likely that incompleteness of the data and loss to follow-up interfered with adequate data collection. A prospective study on patients within the private and public systems in this region is planned, in order to improve the quality of the data.

## CONCLUSION

The characteristics of the patients with inflammatory bowel disease in this study were similar to those encountered in the published literature from Brazil in terms of gender, type of disease and age at diagnosis. On the other hand, higher prevalence of pancolitis than previously described was found. An extension of this project, of prospective nature, may be beneficial with regard to establishing a database with which data from future studies can be correlated and compared.
